# Acute Effects of Ambient Particulate Matter on Mortality in Europe and North America: Results from the APHENA Study

**DOI:** 10.1289/ehp.11345

**Published:** 2008-06-26

**Authors:** Evangelia Samoli, Roger Peng, Tim Ramsay, Marina Pipikou, Giota Touloumi, Francesca Dominici, Rick Burnett, Aaron Cohen, Daniel Krewski, Jon Samet, Klea Katsouyanni

**Affiliations:** 1 Department of Hygiene and Epidemiology, University of Athens Medical School, Athens, Greece; 2 Department of Biostatistics, Johns Hopkins Bloomberg School of Public Health, Baltimore, Maryland, USA; 3 McLaughlin Centre for Population Health Risk Assessment, University of Ottawa, Ottawa, Ontario, Canada; 4 Environmental Health and Consumer Products Branch, Health Canada, Ottawa, Ontario, Canada; 5 Health Effects Institute, Boston, Massachusetts, USA; 6 Department of Epidemiology, Johns Hopkins Bloomberg School of Public Health, Baltimore, Maryland, USA

**Keywords:** air pollution, effect modification, heterogeneity, meta-regression, mortality, natural splines, particulate matter, penalized splines, time-series analysis

## Abstract

**Background:**

The APHENA (Air Pollution and Health: A Combined European and North American Approach) study is a collaborative analysis of multicity time-series data on the effect of air pollution on population health, bringing together data from the European APHEA (Air Pollution and Health: A European Approach) and U.S. NMMAPS (National Morbidity, Mortality and Air Pollution Study) projects, along with Canadian data.

**Objectives:**

The main objective of APHENA was to assess the coherence of the findings of the multicity studies carried out in Europe and North America, when analyzed with a common protocol, and to explore sources of possible heterogeneity. We present APHENA results on the effects of particulate matter (PM) ≤ 10 μm in aerodynamic diameter (PM_10_) on the daily number of deaths for all ages and for those < 75 and ≥ 75 years of age. We explored the impact of potential environmental and socioeconomic factors that may modify this association.

**Methods:**

In the first stage of a two-stage analysis, we used Poisson regression models, with natural and penalized splines, to adjust for seasonality, with various degrees of freedom. In the second stage, we used meta-regression approaches to combine time-series results across cites and to assess effect modification by selected ecologic covariates.

**Results:**

Air pollution risk estimates were relatively robust to different modeling approaches. Risk estimates from Europe and United States were similar, but those from Canada were substantially higher. The combined effect of PM_10_ on all-cause mortality across all ages for cities with daily air pollution data ranged from 0.2% to 0.6% for a 10-μg/m^3^ increase in ambient PM_10_ concentration. Effect modification by other pollutants and climatic variables differed in Europe and the United States. In both of these regions, a higher proportion of older people and higher unemployment were associated with increased air pollution risk.

**Conclusions:**

Estimates of the increased mortality associated with PM air pollution based on the APHENA study were generally comparable with results of previous reports. Overall, risk estimates were similar in Europe and in the United States but higher in Canada. However, PM_10_ effect modification patterns were somewhat different in Europe and the United States.

Hundreds of time-series studies worldwide provide compelling evidence of the health effects of short-term exposure to air pollution. These studies also pose problems of interpretation due to variation in analytic methods and reporting, and the possibility of publication and analytic bias. Meta-analyses of published results can provide information about patterns in the relative rates of mortality and morbidity and evidence as to the causes of their spatial variation, but they inherit many of the same limitations of the individual studies. Coordinated multicity studies, designed partly to address these issues, have now been conducted in Europe and North America (Atkinson et al. 2001; Bell et al. 2004; Burnett and Goldberg 2003; Burnett et al. 1998, 2000; Gryparis et al. 2004; Katsouyanni et al. 1997, 2001; Samet et al. 2000a, 2000b, 2000c) and currently provide the most valid epidemiologic evidence of the effects of short-term exposure. The results of these studies appear broadly similar, but their methods and data characteristics differ, precluding definitive conclusions about their quantitative consistency and about the extent of and reasons for differences in the magnitude of the effects of short-term exposure among regions of the world.

APHENA (Air Pollution and Health: A Combined European and North American Approach) is a collaborative study among investigators involved in the European APHEA (Air Pollution and Health: A European Approach) study (Atkinson et al. 2001; Gryparis et al. 2004; Katsouyanni et al. 1997, 2001) and the U.S. NMMAPS (National Morbidity, Mortality and Air Pollution Study) study (Bell et al. 2004; Samet et al. 2000a, 2000b, 2000c), as well as Canadian studies (Burnett and Goldberg 2003; Burnett et al. 1998, 2000). APHENA addresses the short-term health effects of particulate matter (PM) ≤ 10 μm in aero-dynamic diameter (PM_10_) and ozone on daily mortality and hospital admissions. The project originated at a time when the results of the multicity analyses, including APHEA and NMMAPS, were being reported and considered in the development of ambient air quality standards for PM (European Commission 1999; World Health Organization 2004, 2006). The main objective of the project was to assess the coherence of the findings of the multicity studies carried out in Europe and North America, when analyzed with a common protocol, and to explore reasons for any observed differences in the size of the air pollution relative rates.

In this article, we present the APHENA findings on the association between daily measurements of PM_10_ and mortality. The results, spanning two continents with a wide range of sources of ambient air pollution, are relevant to one of the key uncertainties in our current understanding of the health effects of PM: the identification of those characteristics of PM that are associated with toxicity (National Research Council 2004). Current regulatory standards are based on overall indicators of airborne PM mass as concentration metrics, in the face of uncertainty as to the specific physical and chemical characteristics that determine toxicity. The present study permits exploration of heterogeneity in the effect of PM_10_ on mortality across the broad range of atmospheres included in the APHENA cities.

Any assessment of heterogeneity needs to address the potential consequences of using differing analytic strategies to estimate air pollution health risks, and the extent to which apparent heterogeneity across studies reflects the consequences of different analytical methods. Based on past work by the APHENA investigators and extensive sensitivity analysis, we developed uniform approaches for first-stage (within-city) analyses of the time-series data used in previous reports. We then used the regression estimates in second-stage analyses directed at characterizing heterogeneity of the effect of PM_10_ across the APHENA cities and identifying factors contributing to heterogeneity.

## Materials and Methods

### Data

APHENA was based on previously assembled databases in the first-stage analysis. The database included the 90 U.S. cities in NMMAPS (Samet et al. 2000a, 2000b); the 32 European cities in APHEA, of which 22 had PM_10_ data (Katsouyanni et al. 2001); and 12 larger Canadian cities used in previous multicity projects, selected on the basis of availability of air pollution monitoring data (Burnett and Goldberg 2003; Burnett et al. 1998, 2000).

The databases included daily counts of all-cause mortality [excluding deaths from external causes, according to *International Classification of Diseases, 9th Revision* (World Health Organization 1975), codes > 800] for all ages and by age group (≥ 75 years and < 75 years of age). We obtained air pollution measurements from fixed-site monitoring stations in each city. All Canadian cities and 75 U.S. cities had PM_10_ measurements every 3 or 6 days. All European and 15 U.S. cities had a small number of (apparently random) days on which air pollution measurements were missing. The 15 U.S. cities with full time-series data were representative of the total of 90 U.S. cities with regard to both mortality rates and air pollution levels. We used city-specific time-series data on daily temperature (°C, daily mean) to control for the potential confounding effects of weather. City characteristics both within and between the three collaborating centers (Europe, United States, and Canada) exhibit substantial variability. We provide more details on the data in the Supplemental Material (online at http://www.ehponline.org/members/2008/11345/suppl.pdf).

We restricted analysis to days with PM_10_ concentrations < 150 μg/m^3^, because the relationship between air pollution and mortality within this range is effectively linear (Dominici et al. 2002a; Samoli et al. 2005). This restriction led us to exclude < 2% of the available days in each city, except for three European cities (Erfurt, Prague, and Turin), in which we excluded 3–6% of the total number of available days.

### Methods

When APHENA was initiated, there was ongoing debate about the use of time-series methods to describe the relationship between air pollution and health after Dominici et al. (2002b) and Ramsay et al. (2003) identified modeling issues related to nonparametric smoothing. At that time, questions were raised concerning the choice of smoothing method, the degree of smoothing, and parametric versus nonparametric methods. The U.S. Environmental Protection Agency (EPA) requested that the Health Effects Institute (HEI) organize a systematic reanalysis of selected studies to assess the sensitivity of the original estimates produced with non-parametric modeling strategies, using prespecified alternative modeling approaches. The results were published in a special report (HEI 2003), which concluded that no particular method could be recommended as optimal, and recommended that analysts should incorporate extensive sensitivity analyses to assess the adequacy of control for time-varying potential confounders.

Consequently, the APHENA investigators decided to implement a new protocol for reanalysis of the daily air pollution data from the European and North American cities. First, we fit regression models in each city separately to control for seasonal effects, weather, and other potential confounders. Given simulation results (Peng et al. 2006; Touloumi et al. 2006), we considered two methods to control for confounding: natural splines (NS), for parametric modeling of flexible families of curves (McCullagh and Nelder 1989), and penalized regression splines (PS), as implemented by Wood (2000) in R.

Initial methodological exploration indicated that the number of degrees of freedom (df) for control of seasonality was the most important parameter in model specification with respect to the magnitude of regression coefficient reflecting the effect of air pollution on population health. We carried out extensive sensitivity analyses in which we progressively increased df to control for temporal variation. In the second analytic stage, we used meta-regression to obtain center-specific (Canada, Europe, United States) and overall estimates of risk based on the city-specific risk estimates, and to investigate potential city-level effect modifiers.

### Individual city analysis

We investigated the PM_10_–mortality associations for each city using Poisson regression models allowing for overdispersion. The city-specific model is of the form


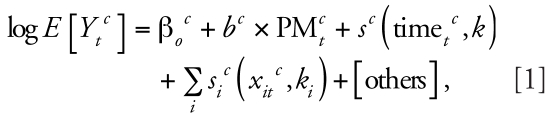


where *E*[*Y_t_^c^*] is the expected value of the Poisson distributed variable *Y_t_^c^* indicating the daily mortality count on day *t* at city *c* with var(*Y_t_^c^*) = ϕE[*Y_t_^c^*], with ϕ representing the overdispersion parameter; PM*_t_^c^* is the air pollution concentration on day *t* in city *c*; and *x_it_^c^* is the value of the *x_i_* meteorologic covariate on day *t* in city *c*. The smooth functions *s* capture the nonlinear relationship between the time-varying covariates and calendar time and daily mortality. We used PS and NS as smooth functions, with *k* denoting the number of basis functions. We used NS as basis functions for the PS. We also included dummy variables for day of the week and holiday effects.

The smooth function of time serves as a proxy for any time-dependent outcome predictors or confounders with long-term trends and seasonal patterns not explicitly included in the model. Hence, we removed long-term trends and seasonal patterns from the data to guard against confounding by omitted covariates. We used 3, 8, and 12 df for seasonality control. Additionally, for complete air pollution time series (i.e., the series without systematic missing values), we used the minimization of the sum of the absolute values of the partial autocorrelation function (PACF) of the model’s residuals as another criterion to select the optimal df, when we applied the PS method. The minimum df allowed under the PACF was 3 df/year. To control for weather, we included smooth terms of temperature on the day of death and the day before death in the time-series models. We used these terms for temperature because in prior analyses we found that same-day temperature accounts for hot-weather effects, whereas previous-day temperature accounts for cold-weather effects. We set the df for both temperature terms at 3, based on an exploratory sensitivity analysis done within APHENA that indicated robust results with respect to different approaches for weather control. For models based on minimization of PACF, we introduced autoregressive terms, if necessary, in case significant autocorrelation remained in the final model’s residuals.

We did not control for influenza epidemics because previously published results have indicated that these do not affect the association between air pollution and mortality (Braga and Zanobetti 2000; Touloumi et al. 2005).

We carried out two sets of analyses, depending on the availability of measurements in each city. We based the first on cities with complete time-series data (i.e., full daily data, with only a few days missing at random), which encompassed all European and 15 U.S. cities. For these cities, we used the average of the same and previous day’s air pollution as a predictor of increased mortality, as well as unconstrained distributed lag models spanning lags 0, 1, and 2. When fitting distributed lag models, we used the same distributed lag terms for temperature as for PM_10_. The second set of analyses included all cities, regardless of data availability, and assessed only the effect of the previous day’s air pollution (lag 1). Because the PACF criterion is based on controlling the autocorrelation in time-series data, we did not apply it when we analyzed the previous day’s air pollution, because this analysis included cities with systematically missing time-series data, for which case the concept of autocorrelation is not straightforward. For complete time series, we then fit eight models (two smoothers and four sets of df for seasonality control) in each city for lags 0 and 1, whereas we applied six models (two smoothers and three sets of df) in cities with systematically missing data for lag 1 analysis.

To investigate potential confounding effects by O_3_, we applied two-pollutant models that controlled for 1-hr maximum O_3_ concentrations.

We also carried out center-specific (Canada, Europe, and United States) threshold analyses to investigate the exposure–response relationship between PM_10_ and all-cause mortality. We used models with NS for confounder control with 8 df per year for seasonality. We selected a grid of threshold values, ranging from 0 to 75 μg/m^3^ in increments of 5 μg/m^3^ (i.e., 0, 5, 10, up to 75 μg/m^3^). For each threshold value *h*, we fit a threshold model to the data for the available cities. In the threshold model, we included a pollutant term (*x*^+^) in the model of the form (pollutant-*h*)^+^, where *x*^+^ = *x* if *x* ≥ 0 and *x*^+^ = 0 if *x* < 0, where *h* is the threshold value. We then computed the Akaike information criterion (AIC) value of the fitted model for all cities in each center for a given threshold value, and then the average AIC for that threshold over all cities in the center. We repeated the analysis for all threshold values. We set a possible threshold at the value that minimized the mean AIC.

### Second-stage analysis

The epidemiologic objectives of the second-stage analysis were to assess bias due, for example, to exposure measurement error; and to assess effect modification of the air pollution relative rates across study regions. Potential effect modifiers used in the analysis included variables describing *a*) the average air pollution level and mix in each city (specifically, the mean levels, standard deviations, and coefficients of variation for PM_10_, nitrogen dioxide, O_3_, and sulfur dioxide and the ratio of PM_10_ to NO_2_); *b*) air pollution level exposure (number of monitors and density of monitors relative to population size); *c*) the health status of the population (cardiorespiratory deaths as a percentage of total mortality, crude mortality rate, directly standardized mortality rate, age structure described as percentage of the population ≥ 65, ≥ 75, and < 15 years of age); and *d*) climatic conditions (mean and variance of temperature and relative humidity levels, and mean minimum and maximum daily temperature). There were few comparable socioeconomic status (SES) indicators across the different countries in Europe at the city level; indeed, only unemployment rate (percent) was available for 14 cities. Unemployment data were available for all U.S. cities.

In the second stage of the analysis, we assumed the city-specific effect estimates, *b^c^*, to be normally distributed around an overall estimate. To test whether variability in *b^c^* was explained by city characteristics, we estimated fixed-effects pooled regression coefficients by weighted regression of *b^c^* on potential effect modifiers (at the city level) with weights inversely proportional to the variances of *b^c^* (DerSimonian and Laird 1986). If we found substantial heterogeneity across cities, beyond the variation associated with the effect modifiers, we applied random-effects regression models. These models assumed that *b^c^* was a sample of independent observations from a normal distribution with the same mean and with variances equal to the between-city variance and the squared SE of *b^c^*. We estimated the random variance component by iteratively reweighted least squares (Berkey et al. 1995).

Based on exploratory analysis, we examined potential effect modification patterns only for cities with complete time-series data and for the effects of the average of 2-day air pollution (lags 0 and 1) because these were more heterogeneous and there were indications that they were relatively insensitive to the choice of analytic method. Because there were differences in the distribution of the effect modifiers between Europe and United States, the cut points for establishing categories for these variables were center specific.

## Results

Table 1 summarizes the center-specific percent increases in the daily number of deaths (all ages and by age group) associated with an increase of 10 μg/m^3^ in PM_10_ concentrations, with and without control for O_3_, estimated by models using 8 df/year and PS, by various lags. (Table 1 presents results from the model using 8 df per year for seasonality control, thus reporting relatively conservative estimates among those from the different modeling strategies applied.) For the Canadian cities, which have measurements for 1 of 6 days, only lag 1 PM_10_ exposure could be considered. Similarly, most U.S. cities had data for 1 of 6 days, so we based the U.S. estimates for lag 1 and longer lags on different numbers of cities (90 and 15, respectively). Air pollution risk estimates for the Canadian cities were about 2-fold higher than those for Europe and the United States. We estimated a lag 1 increase of 10 μg/m^3^ PM_10_ to increase the daily number of deaths by 0.84% [95% confidence interval (CI), 0.30–1.40%] for Canadian cities, 0.33% (95% CI, 0.22–0.44%) for European cities, and 0.29% (95% CI, 0.18–0.40%) for U.S. cities. These estimates decreased slightly with adjustment for O_3_. The effect estimates for people ≥ 75 years of age were consistently larger than those for people < 75 years of age. The previous day’s effects for all ages and for the elderly were statistically significant in all three centers.

When considering the average effect for lags 0 and 1, we estimated an increase of 0.29% (95% CI, 0.14–0.45%) in the daily number of deaths per 10 μg/m^3^ in PM_10_ for European cities and 0.14% (95% CI, –0.12% to 0.40%) for U.S. cities with daily PM_10_ measurements. The effects were higher for the older age group compared with those < 75 years of age. The effects of cumulative exposure, assessed with distributed lag models of lags 0–2, were somewhat lower for European cities than for U.S. cities. PM_10_ effect estimates did not change when controlled for O_3_ levels. When we analyzed the effect of the previous day’s PM_10_ in the 15 U.S. cities with daily time-series data, the corresponding estimates were comparable with those obtained for all 90 U.S. cities, indicating that these 15 cities do not differ systematically from the larger group of 90 cities.

**Figure 1:**
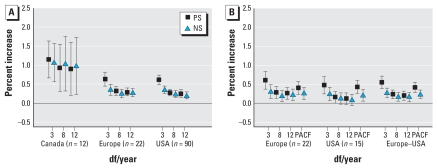
Percent increase in the daily number of deaths, for all ages, associated with a 10-μg/m^3^ increase in PM_10_: lag 1 (*A*) and lags 0 and 1 (*B*) for all three centers. PACF indicates df based on minimization of PACF.

Figure 1 shows the sensitivity of findings to the analytic approach. Figure 1A gives mortality risk estimates for lag 1 PM_10_ concentrations by center. We did not pool the substantially higher estimates for the Canadian cities with those for the U.S. and European cities. There is a tendency for lower estimates to be obtained with greater values of df. Figure 1B shows the results for lags 0 and 1 for cities with daily data (Canadian cities had missing data). The pattern of variation in risk estimates with df was similar to that seen with the lag 1 data. The combined increases in the total number of deaths estimated were 0.25% with PS and 0.18% with NS at 8 df/year, 0.21% with PS and 0.18% with NS at 12 df/year, and 0.42% with PS and 0.25% with NS using the PACF criterion. On average, the PACF method resulted in the selection of 5–6 df per year for seasonality control.

**Figure 2:**
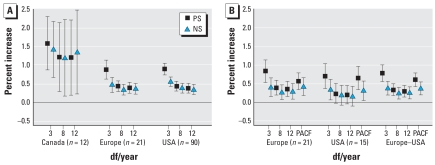
Percent increase in the daily number of deaths, among those ≥ 75 years of age, associated with a 10-μg/m^3^ increase in PM_10_: lag 1 (*A*) and lags 0 and 1 (*B*) for all three centers. PACF indicates df based on minimization of PACF.

**Figure 3:**
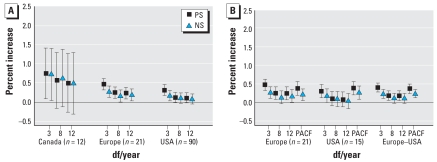
Percent increase in the daily number of deaths, among those < 75 years of age, associated with a 10-μg/m^3^ increase in PM_10_: lag 1 (*A*) and lags 0 and 1 (*B*) for all three centers. PACF indicates df based on minimization of PACF.

Figure 2 shows the effects of PM air pollution on total mortality among persons ≥ 75 years of age. Air pollution risk estimates were higher than those for all ages combined. Because of the difference in effect size for Canada compared with Europe and the United States, we do not provide combined estimates for lag 1. Figure 3 gives the corresponding estimates for those < 75 years of age. Although the size of the PM_10_ effect is smaller, the combined effect for lag 0 and 1 is statistically significant.

The estimated effects of PM_10_ on cardiovascular mortality (data not shown) were generally similar to those for total mortality. Among those ≥ 75 years of age, the effects on cardiovascular mortality were larger than those on total mortality. Specifically, we estimated lag 1 PM_10_ to increase the daily number of cardiovascular deaths among the elderly by 1.30% (95% CI, 0.19–2.40%) in the Canadian cities, 0.47% (95% CI, 0.23–0.70%) in the European cities, and 0.51% (95% CI, 0.29–0.73%) in the U.S. cities. The corresponding estimates for cardiovascular mortality among people < 75 years of age were positive but not significant. There were far fewer respiratory deaths than cardiovascular deaths in all three centers. The results for respiratory mortality were less consistent. PM_10_ at lag 0 and 1 was more consistently associated with increased respiratory mortality, again with larger effects among those ≥ 75 years of age.

We investigated effect modification patterns only for cities with a complete PM_10_ time series. For most of the analytic scenarios considered, the time-series models produced statistically significant effects of PM_10_ on total mortality. However, there was significant heterogeneity in the city-specific estimates of the effects of PM_10_ on total mortality across all ages and among those ≥ 75 years of age. Increasing the df to control for seasonality decreased the magnitude of the air pollution effect and, consequently, the degree of observed heterogeneity. The first-stage results for the European cities were more heterogeneous than those for the U.S. cities. Nevertheless, the European pooled results were more consistent across analytic methods. A detailed presentation of the APHENA second-stage analysis results is available in the Supplemental Material (online at http://www.ehponline.org/members/2008/11345/suppl.pdf).

Effect modification patterns were generally consistent across analytic methods, particularly for those variables having a significant modifying effect on the association between PM air pollution and mortality. The effect modification patterns identified in Europe and the United States were not always consistent. With respect to characteristics of exposure, we found that in Europe higher levels of NO_2_ and a larger NO_2_:PM_10_ ratio were associated with a greater PM_10_ effect on mortality. This pattern was also present in the United States but was less pronounced. In contrast, we saw a smaller PM_10_ effect on mortality among the elderly in cities with higher O_3_ levels, a pattern mainly observed in the U.S. cities. Effect modification by climate was evident in Europe, where higher temperature and lower humidity were associated with larger PM_10_ effects. We found no consistent pattern of effect modification with temperature in the United States, and the association with humidity tended to be inverse. When we investigated variables characterizing the age structure and health status of the population, an increasing proportion of elderly people was associated with higher PM_10_ effects in both Europe and the United States. A larger proportion of cardiorespiratory deaths among all deaths was associated with higher PM_10_ effects only in the United States, and there only among the elderly. The corresponding pattern in Europe was nonsignificant and tended to be the inverse. A higher crude mortality rate was associated with a higher PM_10_ effect in the United States. The only socioeconomic factor available for all cities was the percentage of unemployed: a higher percentage of unemployed was associated with greater PM air pollution effects on both continents.

Investigation of the exposure–response relationship between PM_10_ and total mortality across all ages in APHENA did not support the presence of a threshold in any of the three centers. If a threshold were present, we would expect to see a U-shaped curve when we plot the AIC values for the various threshold models against the thresholds used, with the minimum AIC value corresponding to the threshold. In fact, within each center, the city-specific AIC plots were quite flat for most cities (data not shown).

## Discussion

In this article, we report the results of a comprehensive analysis of time-series data relating PM air pollution to mortality in the general population in 124 cities in Europe (22 cities), the United States (90 cities), and Canada (12 cities). The analysis protocol used in the APHENA study was informed by theoretical developments, sensitivity analyses, and simulations, which we then used to complete a comprehensive reanalysis of time-series data from Europe and North America. Overall, using this common protocol, PM_10_ was associated with increased total mortality, particularly among those ≥ 75 years of age, in all three centers (Europe, United States, and Canada), with the effect notably greater in Canadian cities. Mortality risk estimates tended to decrease with increasing adjustment for unmeasured time-varying covariates and were generally lower for the average of lags 0 and 1, compared with lag 1 alone. Distributed lag models exhibited a different risk pattern between the United States and Europe, with a more prolonged effect of exposure to PM_10_ seen in the United States.

The effects of PM_10_ on total mortality in European and U.S. cities were quite similar. Based on different modeling approaches, results from the same data sets have been previously reported and were quite close, with the small discrepancies noted possibly due to the differing modeling approaches. For the European cities, one APHEA report (Katsouyanni et al. 2001) provided an estimate of 0.6% increase in the daily total number of deaths per 10 μg/m^3^ PM_10_, whereas the reanalysis provided an estimate of 0.4% (HEI 2003). Similarly, original NMMAPS results reported in Samet et al. (2000b) estimated an increase in the number of deaths of 0.4%, and the reanalysis reported a 0.2% increase (HEI 2003) based on 90 U.S. cities. Within the context of APHENA, we had the opportunity to expand the investigation of within-center heterogeneity previously reported. The main effect modification patterns identified by Katsouyanni et al. (2001) were replicated within APHENA, and several new modifiers were identified as well—for example, the modifying effect of the percentage of unemployed on the association between PM air pollution and mortality. The significantly higher estimates observed in Canada previously (0.8%; HEI 2003) persisted in the present APHENA analysis.

Because we analyzed the data according to standardized criteria in APHENA, the higher values observed in Canada cannot be attributed to differences in analytic approaches. Nevertheless, city-specific estimates of the effect of PM_10_ on mortality for Canadian cities such as Toronto (in which mortality among the elderly was increased by 1.4% for a 10-μg/m^3^ increase in PM_10_) and U.S. cities of similar population size and climate such as Detroit, Michigan (0.8% mortality increase), were close. The trend toward higher estimates could possibly be the result of more accurate exposure and outcome data in Canada compared with the European countries and the United States. (At this point, we have no validation data available to explore this possibility.) Although the effect of PM_10_ on mortality may be greater in Canada compared with the other countries, we cannot readily identify any specific source mix differences among the APHENA countries that might explain this difference. Alternatively, although no threshold has been detected in the exposure–response association between ambient PM and mortality, there may be a log-linear association between air pollution and mortality, for which lower pollution levels contribute larger risks; under this hypothesis, the lower air pollution concentrations in Canadian cities would lead to higher risks. An additional explanation, which cannot be explored with the APHENA data, would be that PM_10_ acts primarily as a surrogate of the true causal pollutants and that the relationship between PM_10_ and the toxic components differs in Canada compared with the other countries.

Several meta-analyses of the effect of PM_10_ on mortality have been reported (Anderson et al. 2004, 2005; Pope and Dockery 2006; Stieb et al. 2002, 2003). The combined estimates from single-city studies tend to be higher, partly because not all estimates have been revised subsequent to the identification of the S-Plus convergence criteria issue (Anderson et al. 2005). Furthermore, aspects of city selection and model specification in the single-city studies may have led to upwardly biased estimates; there is also some evidence of publication bias, which would also tend to result in an upward bias (Anderson et al. 2005). Summary estimates from single-city studies range from about 0.4% to 0.8% per 10 μg/m^3^ PM_10_ (Pope and Dockery 2006). The European and U.S. estimates in APHENA lie just below this range, whereas the Canadian estimates are at the upper end of the range.

European cities tend to have a higher prevalence of diesel vehicles, particularly passenger cars, than do cities in North America (European Commission 2005; Green Car Congress 2008); although not characterized, source inventories related to power generation and industry are also likely to vary, both between and within continents. The comparability of European and U.S. risk estimates suggests that underlying differences in PM air pollution sources may not have substantially affected the overall risk.

One objective of APHENA was to explore patterns of effect modification across a wide range of geographic locations with air pollution coming from differing source mixtures and with populations differing in sociodemographic characteristics.

In prior analyses of both single-city and multicity data, a number of potential modifiers of associations between air pollution and mortality have been identified (O’Neill et al. 2003; U.S. EPA 2004). Within APHEA, prior analyses identified modification of the effect of PM_10_ on both mortality and admissions outcomes (Aga et al. 2003; Analitis et al. 2006; Atkinson et al. 2001; Katsouyanni et al. 2001; Le Tertre et al. 2002; Samoli et al. 2005). In NMMAPS, Samet et al. (2000a, 2000c) explored effect modification extensively in the original analyses of the 90 cities’ mortality data and identified several potential modifiers. Both projects found evidence of variation by geographic region. Similar two-stage analyses have not been carried out previously for the Canadian cities.

We addressed effect modification in APHENA in the second-stage analysis using city-level variables indicative of characteristics of the air pollution mixture, climate, age structure and health status, and SES determinants. PM_10_ effect modification patterns, explored only for cities with daily data (21 European and 15 U.S. cities), were not entirely consistent across centers and varied somewhat depending on the underlying model and geographic location. Pollutant levels demonstrated different modifying effects for cities in Europe and the United States, which may be attributed to variation in the complex mix of air pollutants in Europe and the United States. Climatic variables were important only in Europe. One explanation may be related to the lower prevalence of air conditioning in Europe, which would lead to a higher exposure of the population to outdoor air in indoor environments. We found the most consistent evidence of effect modification for age, with an increasing proportion ≥ 75 years associated with a greater effect of PM_10_ in both the United States and Europe; higher percentage of unemployment was also associated with greater risk in both continents.

Larger proportions of persons ≥ 75 years of age were associated in both centers with larger PM_10_ effects on mortality all ages and in this age group. This finding may be associated with lower baseline mortality, possibly leading to higher relative risks. Another plausible explanation may be that in locations with larger proportions of those ≥ 75 years of age, the mean age of this group is also larger, leading to an excess effect. The positive effect modification pattern associated with higher unemployment suggests that populations with lower SES may be more susceptible to PM_10_, as was noted by Krewski et al. (2000) in studies of the effects of long-term exposure to PM_2.5_ on mortality.

The proportion of cardiopulmonary deaths relative to the total number of deaths displayed an opposing pattern in European and U.S. cities. In Europe, each national authority was responsible for coding the causes of death, and that the comparability of this practice has not been evaluated.

The principal limitations in interpreting the APHENA findings lie with the data available to the investigators. The data came from multiple countries and were not collected according to a uniform protocol. Although we edited and analyzed all data extensively and used a quality assessment audit, differing measurement error structures across the three sets of data remains a possible source of heterogeneity.

Although a number of potential effect modifiers have been identified, the exploration of effect modification in APHENA was limited by the restricted number of variables that extended across the full data set. Finally, the relatively small number of cities with daily data and the large statistical uncertainty of the city-specific estimates may have limited the power for detecting effect modification patterns. A more thorough discussion on the limitations of APHENA is available in the Supplemental Material (online at http://www.ehponline.org/members/2008/11345/suppl.pdf).

The APHENA study led to the development of a standardized protocol for analyses of daily time-series data on air pollution and mortality. We pooled data from studies that had been carried out in multiple cities in Europe and North America. The findings confirm the acute, adverse effects of PM_10_ on mortality. The use of the primary raw data to conduct pooled analyses in APHENA permits more informative analysis than can be achieved through a simpler meta-analysis of summary risk estimates taken from the published literature. Despite the differences in data availability and comparability of effect modifiers in Europe and North America, it is possible to consolidate an ongoing collection of time-series data into a single data set so that similar analyses can be carried out periodically in the future. For the purpose of air pollution control, a need exists for periodic syntheses of the literature to serve as the basis for establishing air quality guidelines at the national and international level (Craig et al. 2008); thus, there is a strong rationale for the ongoing collection and synthesis of estimates of the health risks of air pollution. This rationale is particularly compelling for regions of the world where levels of air pollution are higher and population susceptibility may be increased, as well (e.g., Asia and Latin America). APHENA has already served as an example and a source of methodological guidance for two ongoing coordinated multicity studies: PAPA (Public Health and Air Pollution in Asia) and ESCALA (Estudio de Salud y Contaminación del Aire en Latinoamérica) (Gouveia et al. 2008; Wong 2008).

## Figures and Tables

**Table 1:** Percent increase (95% CI) in the daily number of deaths (all ages and = 75 and < 75 years of age) associated with an increase of 10 μg/m^3^ in PM_10_ concentrations (estimated by using 8 df/year to control for seasonal patterns and PS) in the three centers.

	Total mortality			
Age group/center	Lag 1	Controlling for O_3_ (lag 1)	Average of lags 0, 1	Distributed lag models (lags 0, 1, 2)
All ages (years)				
Canada	0.84 (0.30 to 1.40)	0.76 (0.20 to 1.30)	NA	NA
Europe	0.33 (0.22 to 0.44)	0.32 (0.21 to 0.42)	0.29 (0.14 to 0.45)	0.20 (–0.01 to 0.42)
United States^a^	0.29 (0.18 to 0.40)	0.24 (0.08 to 0.41)	0.14 (–0.12 to 0.40)	0.26 (–0.08 to 0.61)
≥ 75 years				
Canada	1.00 (0.25 to 1.80)	0.98 (0.18 to 1.80)	NA	NA
Europe	0.44 (0.29 to 0.58)	0.41 (0.27 to 0.54)	0.39 (0.19 to 0.59)	0.32 (0.04 to 0.60)
United States^a^	0.47 (0.31 to 0.63)	0.37 (0.16 to 0.59)	0.19 (–0.19 to 0.56)	0.33 (–0.16 to 0.82)
< 75 years				
Canada	0.63 (–0.12 to 1.40)	0.51 (–0.26 to 1.30)	NA	NA
Europe	0.25 (0.10 to 0.40)	0.23 (0.07 to 0.39)	0.25 (0.09 to 0.42)	0.11 (–0.20 to 0.43)
United States^a^	0.12 (–0.02 to 0.27)	0.10 (–0.13 to 0.34)	0.09 (–0.20 to 0.38)	0.20 (–0.24 to 0.63)

Abbreviations: CI, confidence interval; NA, not applied because of systematically missing data.

a We based the U.S. estimates for lag 1 on 90 cities, and the average of lags 0 and 1 and distributed lag models on 15 cities.
